# A systematic review to assess current surface water and sediment microplastic sampling practices in seagrass and mangrove ecosystems

**DOI:** 10.1007/s11356-024-35690-9

**Published:** 2024-12-11

**Authors:** Jack Greenshields, Amie Anastasi, Andrew D. Irving, Angela Capper

**Affiliations:** 1 Coastal Marine Ecosystems Research Centre, Central Queensland University, Gladstone, 4680 Australia; 2 Central Queensland Innovation and Research Precinct, Central Queensland University, Rockhampton, 4701 Australia

**Keywords:** Plastic pollution, Microplastics, Standardised methodology, Coastal ecosystems

## Abstract

Global plastic production is estimated to be 400 million tonnes per annum, with ~ 5.25 trillion fragments floating in our oceans. Microplastics (< 5 mm) have the potential to disproportionately accumulate and become trapped in mangroves and seagrass meadows, creating plastic ‘sinks’. This is concerning as these ecosystems are of great ecological and economic importance, with microplastics causing harm to inhabiting flora and fauna. However, accurately measuring microplastic abundance, comparing findings, and determining potential impacts are difficult due to a lack of standardised sampling protocols. Therefore, a systematic literature review was completed to review currently adopted microplastic sampling methods in surface water and sediment in seagrass and mangrove ecosystems. These were compared with recommendations from existing governmental and institutional groups as a first step to standardising methods for future sampling procedures in seagrasses and mangroves.

**Supplementary Information:**

The online version contains supplementary material available at 10.1007/s11356-024-35690-9.

## Introduction

Global production of plastics by industry rose to 400 Mmt per annum in 2022 (Geyer et al. 2017; Plastics Europe 2023). Mismanaged plastic waste accumulates in the ocean, largely from land-based sources (~ 80%), including stormwater drains, catchment run-off, and wastewater processing (UNEP 2016). Current estimates place ~ 5.25 trillion floating plastic fragments, with 12.7 Mmt being added each year (Jambeck et al. 2015; Xanthos & Walker 2017). While these figures are alarming, accurate sampling methodologies are relied upon to create these estimates, which are widely disseminated to global audiences. However, with a lack of standardised methods for sampling plastic pollutants, including microplastics (< 5 mm), concerns for associated impacts are compounded by a lack of comparable data.

Floating plastics are present in a range of aquatic ecosystems, including coastal habitats such as seagrasses and mangroves. These ecosystems have unique canopy and root structures that may cause microplastics to disproportionately accumulate, forming plastic ‘sinks’. Microplastic concentrations in seagrasses and mangroves worldwide vary considerably, being influenced by factors including proximity to urban areas, tidal dynamics, and sampling methodologies. In mangrove sediments, concentrations range from 1.22 to 6390 particles per kilogram of dry sediment, while surface water samples show lower concentrations, between 3.75 and 477 particles per cubic metre (Maghsodian et al. 2022). For seagrasses, sediment concentrations vary from 0 to 1817 particles per kilogram of dry sediment, and surface waters contain between 0.04 and 94 particles per litre (Jeyasanta et al. 2020; Kreitsberg et al. 2021). Significantly higher microplastic accumulation has been recorded in sediment sampled from seagrass beds and mangroves compared to bare sediment (Deng et al. 2020; Jones et al. 2020; Zhao et al. 2022), and even encrusting on leaves (Goss et al. 2018; Li et al. 2022), while other studies have observed no such effect (Boshoff et al. 2023; Unsworth et al. 2021). These conflicting findings highlight the under-explored dynamics of microplastic pollution in seagrasses and mangroves (Ciaralli et al. 2024; Deng et al. 2021), as well as identifying a lack of commonality within sampling, use of units, and reporting methods.

The accumulation of microplastics in these ecologically essential habitats is cause for concern, as they not only provide essential nursery habitats for a wide range of recreationally and commercially important species (Bertelli & Unsworth 2014), they also minimise coastal erosion and are a vital component in the ‘blue carbon’ solution (d’Avack et al. 2019; Huxham et al. 2018). Consumption of microplastics by a range of inhabiting species from micro- to mega-grazers can exert harmful physiological effects, with absorbed toxins and harmful plastic additives potentially biomagnificating through food webs to harm marine life at higher trophic levels (Bonanno & Orlando-Bonaca 2018). Aggregation of microplastics in the water and sediment may not only harm resident fauna, but also the seagrasses and mangroves themselves. Molin et al. (2023) observed a ~ 50% decline over 14 days in the dark respiration rate and a ~ 30% decline in maximum photosynthetic rate of *Zostera marina* subjected to 1000 mg microplastics L^−1^ released into seawater compared to controls. In mangroves, polyethylene and polypropylene microplastics can reduce root and leaf biomass in *Kandelia obovata*, in addition to lowering chlorophyll concentration and photosynthetic efficiency (Chai et al. 2023). Therefore, obtaining comparable microplastic data using standardised methods is essential for effective protection efforts.

The number of studies assessing the impacts of microplastic accumulation in these important ecosystems is increasing, though a lack of standardised protocols for sampling methodologies hinders comparability. Conflicting methodologies between studies may contribute to contradictory results regarding the microplastic trapping capability of these ecosystems (Unsworth et al. 2021; Watkins et al. 2021). Therefore, this review aims to (1) conduct a systematic literature review to identify the most widely adopted microplastic sampling methods for surface water and sediment in seagrasses and mangroves; (2) compare currently utilised collection methods and sampling parameters against current governmental and institutional microplastic sampling protocols; and (3) propose protocols to aid in standardising sampling procedures for future microplastics research in seagrasses and mangroves.

## Review methodology

The literature published between Jan 2014 and Apr 2024 was reviewed using Web of Science (all databases) as a formal scientific library. This was then supplemented by the first 200 results of a Google Scholar search for a more robust primary literature search as well as grey literature as recommended by Haddaway et al. (2015). Primary literature was defined as research papers, while grey literature was defined as government and industry reports, conference proceedings, and any other internally or non-commercially produced documents. Only experimental papers were included. Review papers, studies on macroplastics or nanoplastics, and studies that collected media purely for additive or bacterial extraction were excluded.

**Fig. 1:**
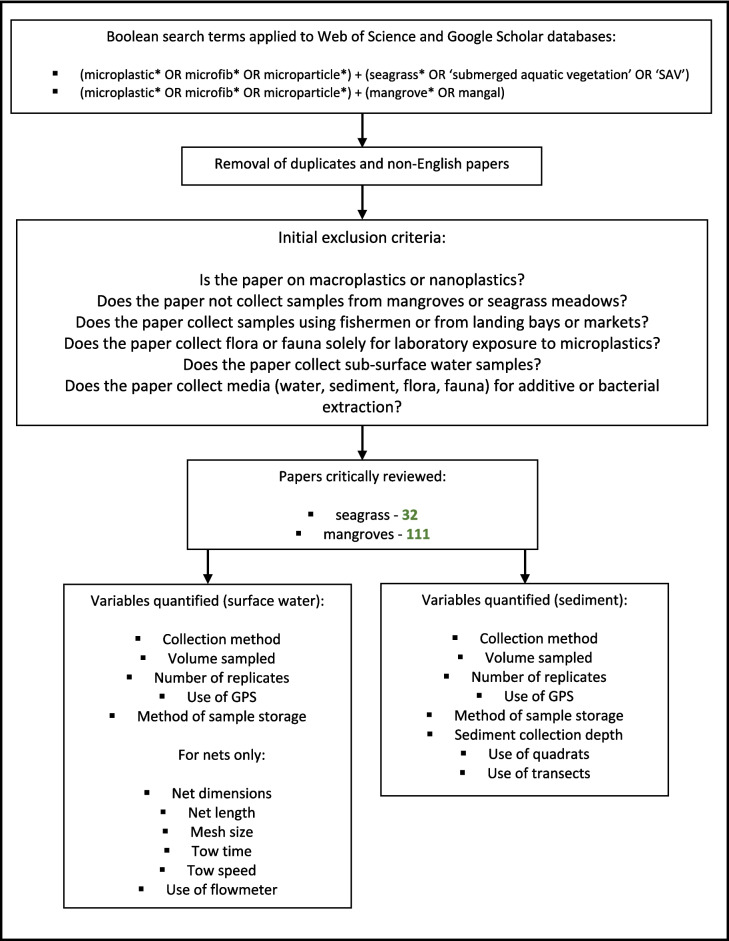
Flow chart of systematic review structure, including search terms, exclusion criteria, and variables quantified for analysis

**Fig. 2:**
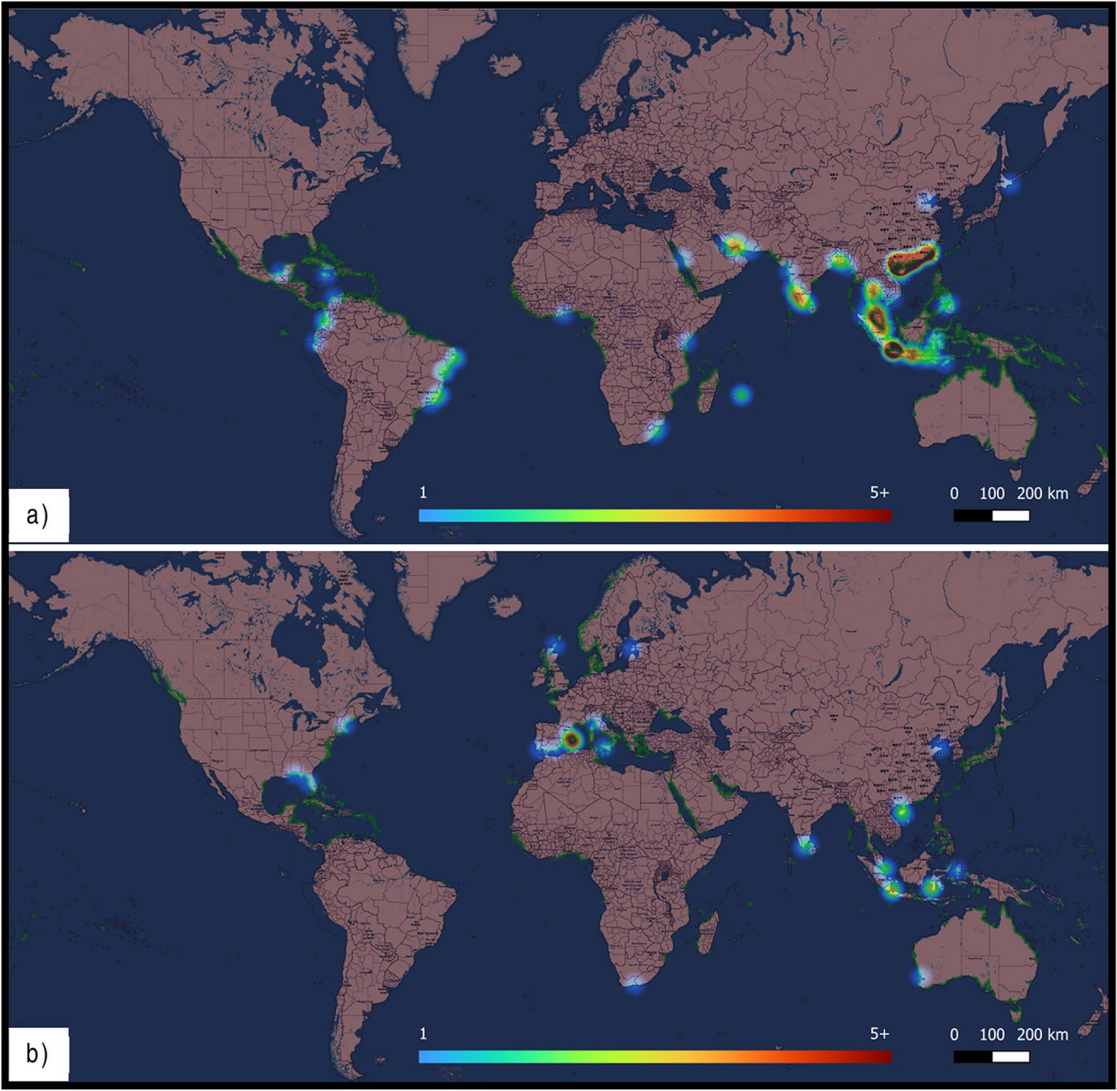
Heatmap identifying microplastic study locations in (**a**) mangroves, with the global distribution of mangroves overlayed in green (Bunting et al. 2022); and (**b**) seagrass beds, with the global distribution of seagrass overlayed in green (UNEP-WCMC Short FT 2020). The colour scale from 1 (low density) to 5 + (high density) indicates the density of studies in a given location

The research resulted in 32 seagrass papers and 111 mangrove papers being accepted for review. All articles used in this review are shown in the supplementary material (Table S1). Selected articles were thoroughly screened to collect data for surface water and sediment sampling. For net surface water sampling, manta and neuston nets were assumed to have floats attached unless otherwise specified. A workflow to demonstrate systematic review search terms, steps, Boolean terms, applied exclusion criteria, and variables quantified for analysis is detailed in Fig. 1. In addition, using QGIS (version 2.26.2), two spatial heatmaps were created to represent the spatial distribution of seagrass and mangrove microplastic research in both intertidal and subtidal environments. Initially, geographic shapefiles were imported to visualise the global distribution of seagrasses and mangroves (Bunting et al. 2022; UNEP-WCMC Short FT 2020). GPS locations of selected articles were then overlayed as respective heatmaps to compare the density of sampled areas with the worldwide distribution of these ecosystems (Fig. 2).

## Results

### Mangrove and seagrass studies vs. global coverage

The combined mangrove and seagrass studies identified in this review spanned a total of 26 countries. Mangrove studies encompassed 21 countries, with research clustered in the Indo-Pacific region, specifically in China (*n* = 34) and Indonesia (*n* = 23). Other countries with high output include India (*n* = 10), Thailand (*n* = 7), Brazil (*n* = 7), and Malaysia (*n* = 6). Seagrass studies encompassed 12 countries, with high levels of clustering around Indonesia (*n* = 8) and Spain (*n* = 7). Other countries with higher output include China (*n* = 4) and the USA (*n* = 3). The scarcity of microplastic studies when compared to these extensive global habitats can be observed (Fig. 2). In particular, Central America, Northern Europe, Western Africa, and Oceania are under-represented.

### Surface water sampling variables in seagrass and mangrove ecosystems

**Fig. 3:**
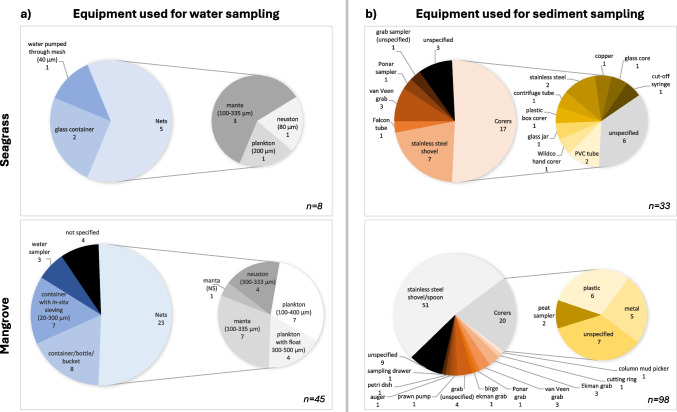
Systematic literature search outlining sampling equipment used for microplastic collection in (**a**) the surface water and (**b**) the sediment of seagrass meadows and mangroves. Each number refers to the number of publications which utilised this method

From the 51 reviewed papers that conducted surface water sampling for microplastics, nets were the most commonly used equipment in both seagrass meadows (63% of studies) and mangroves (50%). When sampling in seagrass meadows, the most popular net type was manta, while plankton nets were most common in mangroves (Fig. 3a). Mesh sizes ranged from 80 to 500 µm (Expósito et al. 2021; Garcés-Ordóñez et al. 2021), with 330–335 µm most frequently used in 57% of studies (Table 1).

**Table 1:** Frequency of net types and mesh sizes used in microplastic surface water sampling in mangroves and seagrasses. *NS*, not specified

Net type	Mesh size (µm)	Frequency
Manta	NS	1
Manta	100	2
Manta	300	2
Manta	330	3
Manta	333	1
Manta	335	2
Neuston	80	1
Neuston	330	3
Neuston	333	1
Plankton	100	1
Plankton	150	1
Plankton	200	3
Plankton	300	4
Plankton	400	1
Plankton	500	2

When using a boat to tow (75%), net aperture width ranged from 24 to 120 cm (Aliabad et al. 2019; Expósito et al. 2021), with 60 cm being the most common (43%). Net length ranged from 0.75 to 3 m (Al Nahian et al. 2022; Kama et al. 2021), with 1 m being the most common length in seagrasses, and 3 m in mangroves (Tables 2 and 3). However, 65% of studies did not specify at least one piece of this information (mesh size, aperture size, or net length). Tow time ranged from 5 to 180 min (Lins-Silva et al. 2021; Tan & Mohd Zanuri 2023). Tow speed ranged from 1 to 5 knots (Jeyasanta et al. 2020; Rose & Webber 2019), with 2–3 knots being the most common (52%). Forty-eight percent of studies used a flowmeter to calculate sample volume while towing. In addition to nets, 43% of studies used a bottle or container to collect water samples that were also used for storage. Thirteen percent of studies used containers with in situ sieving, 6% used a water sampler, 2% used a boat water pump, and 8% did not specify their method of collection. The number of replicates ranged from 1 to 6 when using a boat to tow a net (Jeyasanta et al. 2023; Kama et al. 2021), with one replicate being used in 62% of studies. When using containers, 1–7 replicates were used (Jitrapat et al. 2024; Kannankai et al. 2022). When specified, the amount of water collected per container ranged from approximately 166 to 3000 mL (Kannankai et al. 2022; Wei et al. 2022). The most common method of storage was glass bottles, which occurred in 32% of studies.

**Table 2:** Frequency of aperture sizes used by different net types in microplastic surface water sampling in mangroves and seagrasses. *NS*, not specified

Net type	Net aperture size (cm)									
	24	30	45	50	58	60	70	100	120	NS
Manta	-	1	1	1	1	3	1	1	-	1
Neuston	1	-	-	-	-	2	-	-	1	1
Plankton	-	1	-	-	-	4	-	-	-	1

**Table 3:** Frequency of lengths used by different net types in microplastic surface water sampling in mangroves and seagrasses. *NS*, not specified

Net type	Mangrove/seagrass	Net length (m)								
		0.75	1	1.5	1.8	2	2.5	2.6	3	NS
Manta	M S	- -	- 1	1 -	1 -	1 -	- -	- -	2 1	3 -
Neuston	M S	1 -	- 1	- -	- -	- -	1 -	- -	- -	2 -
Plankton	M S	- -	- -	- -	- -	- -	- -	1 -	- -	5 -

### Sediment sampling variables in seagrass and mangrove ecosystems

From the 126 papers reviewed that conducted sediment sampling for microplastics, corers were the most widely used equipment to sample seagrass bed sediments (52%), with stainless steel shovels/spoons most used in mangroves (52%, Fig. 3b). When using corers, sampling depth ranged from 3 to 170 cm (Cozzolino et al. 2020; Martin et al. 2020), with 5 cm being utilised most often in 20% of studies. Sampling volume ranged from 35 to 5216 cm^3^ (Martin et al. 2020; Plee & Pomory 2020), with 1–72 replicates (Dung et al. 2021; Wright et al. 2023; Tables 4, 5, 6). Sampling depth for shovels/spoons ranged from 1 to 33 cm (Paes et al. 2022; Zhang et al. 2020), with the most common being 5 cm (43%). Sampling volume ranged from 3 to 3000 cm^3^ (Maghsodian et al. 2021; Naji et al. 2017), with 1–21 replicates (Kumkar et al. 2021; Zuo et al. 2020). Grabs were utilised in 13% of studies, with Van Veen grabs the most common (41%). Sampling depth for grabs ranged from 2 to 23 cm (Ibrahim et al. 2021; Ni’am et al. 2022), with 5 cm the most common depth in 41% of studies. Sampling volume ranged from 400 to 2000 cm^3^ (Fatema et al. 2023; Ibrahim et al. 2021), with 1–3 replicates (Fatema et al. 2023; Jeyasanta et al. 2023; Tables 4, 5, 6). However, 65% of studies failed to provide at least one factor of this key information. In addition, 18% of studies used transects, and 36% of studies used quadrats to stratify their sampling. Quadrats sizes of 50 × 50 cm were the most frequently used (22%). The most common storage method was an aluminium box/foil, which occurred in 25% of studies, though 38% did not specify.

**Table 4:** Frequency of sampling depths used by different equipment for microplastic sediment sampling in mangroves and seagrasses. *NS*, not specified

Equipment	Sample depth (cm)														
	1	2	3	4	5	6–9	10	11–15	16–29	30	31–50	51–99	100	101–170	NS
Corer	-	-	1	-	8	2	7	1	1	7	1	-	5	2	5
Grab	-	1	-	-	7	-	1	-	2	-	-	-	-	-	6
Shovel/spoon	1	9	7	3	26	3	6	2	-	-	1	-	-	-	2

**Table 5:** Frequency of sampling volumes collected by different equipment for microplastic sediment sampling in mangroves and seagrasses. *NS*, not specified

Equipment	Sample volume (cm^3^)									
	1–9	10–49	50–99	100–249	250–499	500–999	1000–1999	2000–2999	≥ 3000	NS
Corer	-	2	3	4	5	5	3	1	4	10
Grab	-	-	-	-	1	2	2	2	-	10
Shovel/spoon	1	1	1	3	6	5	13	5	2	21

**Table 6:** Frequency of replicates collected by different equipment for microplastic sediment sampling in mangroves and seagrasses. *NS*, not specified

Equipment	Replicates								
	1	2	3	5	6–9	10–15	16–29	≥ 30	NS
Corer	6	6	10	5	1	1	1	1	6
Grab	3	2	6	-	-	-	-	-	6
Shovel/spoon	12	5	25	2	5	1	1	-	7

## Discussion

### Surface water sampling in seagrass and mangrove ecosystems

#### Comparisons with current governmental and institutional guidelines

Multiple governmental groups have provided recommendations for sampling microplastics in water. These microplastic sampling methods were originally developed to study species abundance and distribution of marine plankton in ecosystem health assessments, and were repurposed for microplastic assessment in coastal waters. Therefore, they do not specifically account for the additional challenges pertaining to sampling within mangrove and seagrass ecosystems such as tidal variation, wave action, and access difficulties. However, these guidelines provide a baseline for standardised sampling protocols in these ecosystems. These include the European Union Marine Strategy Framework Directive (MSFD) technical group (MSFD Technical Group on Marine Litter 2023; MSFD technical subgroup 2013), the National Oceanic and Atmospheric Administration (NOAA) (NOAA 2015), and the Joint Programming Initiative Healthy and Productive Seas and Oceans (JPI Oceans) BASEMAN group (Frias et al. 2018; Gago et al. 2018).

The MSFD technical subgroup (2013) recommends the use of a manta trawl, due to its ability to deploy in a range of sea states, which is useful when sampling closer inshore where weather conditions are usually less calm. In addition, high-speed manta trawls can be efficient at up to 8 knots, allowing large volumes of water to be sampled. Unfortunately, to reach these towing speeds larger mesh sizes need to be used, rendering these designs relatively insensitive to smaller size fractions. They also recommended a 333 µm mesh size and 6 m length to promote the highest inter-comparability among sampling programs in their 2013 report. An updated report published in 2023 amended their mesh size recommendations to 300 µm (MSFD Technical Group on Marine Litter 2023). NOAA (NOAA 2015) suggests the use of a 335-µm manta trawl, while the JPI Oceans BASEMAN group (Gago et al. 2018) advises either a manta or neuston trawl. Recommended towing durations range from 10 to 30 min, though all groups note that factors such as plankton blooms clogging nets make it difficult to set a standardised haul duration.

None of the above three groups recommends the use of a plankton net. This conflicts with the results seen from this systematic review, as plankton nets were the most common microplastic sampling equipment used (Fig. 3a). Of the three types of net evaluated in this study, plankton nets are the smallest and most inexpensive; and due to their myriad of uses, many research institutions and universities stock them. However, as no reviewed studies explicitly justified their net preferences, the reasoning behind specific choices can only be speculated.

In summary, the majority of the aforementioned governmental and institutional guidelines for surface water sampling for microplastics recommend the use of a manta trawl with a 300–335 µm mesh. In this systematic review, manta trawls accounted for only 39% of the total net types used, with only 21% of studies using a 300 µm mesh size as recommended by the MSFD technical group 2023 report (MSFD Technical Group on Marine Litter 2023), and only 14% of studies using mesh sizes of 333–335 µm as recommended by MSFD technical subgroup (2013) and NOAA (2015). This variability highlights a fundamental issue affecting direct comparison of data among microplastic studies in seagrasses and mangroves to date, necessitating an informed agreed-upon method with more standardised parameters. To aid this, the advantages and disadvantages of each sampling type observed in the literature for microplastic collection for surface waters are summarised in Table 7.

**Table 7:** Equipment-specific advantages and disadvantages of surface water sampling methods utilised in seagrass meadows and mangroves. Adapted from Prata et al. (2019)

Equipment	Advantages	Disadvantages
Manta net	Variants can be towed at high speed (8 knots) Samples large volumes of water High stability, can be deployed in a range of sea states Tows at set water depth	Expensive Requires a boat Larger mesh size (≥ 333 µm) for higher tow speeds Possible contamination from equipment and vessel
Neuston net	Usually largest aperture of all net types Samples large volumes of water	Expensive Requires a boat Water depth sampled determined by sea state Design requires calmer sea states than manta trawl Lower speeds than manta trawl Possible contamination from equipment and vessel
Plankton net	Least expensive of the three net types Commonly lowest detection limits (100 µm)	High chance of clogging Samples lower water volumes than other net designs Lower tow speeds than manta and neuston Requires continuous water flow No set water depth (unless attaching float) Possible contamination from equipment
Pumps	Can sample large volumes of water easily Choice of mesh size to filter	Requires additional equipment and energy Possible contamination from equipment and vessel
Sieving	Inexpensive Boat not required Easy transportation	Laborious and time-consuming Sample volume not as large as pumps or nets
Samplers/jars/containers	Inexpensive Boat not required Choice of mesh size to filter Can be used in difficult-to-reach areas	Small samples may be unrepresentative of area Potentially non-standardised sampling depth Laborious and time-consuming Transport of water samples Possible contamination if using plastic equipment

#### Standardising future surface water sampling

Advantages and disadvantages exist for each surface water sampling method (Table 7). The choices made by researchers regarding sampling are influenced by factors such as funding, site availability, and access. However, considering these factors, protocol suggestions can be made as an initial step towards more standardised microplastic sampling practices in the surface waters of seagrass meadows and mangroves.

Manta trawl nets are recommended over other net types (i.e. neuston and plankton) for microplastic sampling for subtidal locations in seagrass and mangrove ecosystems. Due to mangroves and seagrass meadows being near-shore ecosystems, they experience high wave action and tidal variation. The manta net design provides the highest stability in rougher conditions and has a set sampling depth irrespective of wave action, which allows for a greater degree of standardisation among studies. Over a decade ago, Hidalgo-Ruz et al. (2012) reported that the most common mesh sizes used for surface water microplastic sampling were between 300 and 390 µm. This recommendation still stands and concurs with the findings of this research, as the most common mesh sizes observed were between 330 and 335 µm. However, Lindeque et al. (2020) observed that using a 100 µm net yielded a 2.5-fold increase in microplastics collected compared to a 333 µm net, and a tenfold increase compared to a 500 µm net. Only 7% of studies in this review used meshes of 100 µm or below. Therefore, although current mesh sizes in the literature are in line with guidelines from NOAA (NOAA 2015) and the MSFD technical group (MSFD Technical Group on Marine Litter 2023; MSFD technical subgroup 2013), future research activities should switch to finer mesh (100 µm or below) as this smaller mesh size would provide a more accurate approximation of microplastic abundance in these and other ecosystems.

There is a lack of evidence in the current microplastic literature indicating the efficacy of different dimensions of manta net aperture, width, or net length in capturing microplastics. In the absence of this data, it is particularly important as a research collective to standardise these parameters as much as possible to facilitate meaningful comparisons among datasets. From the studies reviewed and the collective of guidelines published thus far, a net aperture width of 60 cm, a depth of 25 cm, and a length of up to 3 m are recommended. Aperture dimensions for manta nets are set at approximately 60 cm by various manufacturers (5 gyres institute 2020; Aquatic BioTechnology 2021; NHBS 2021), and were also the most common aperture size observed in this systematic review (43% of studies). Average net depth of the reviewed studies was set at approximately a third of the aperture width to maintain stability and speed while towing. The MSFD technical subgroup (2013) recommends a net length of 6 m; however, in mangrove and seagrass ecosystems where water can be relatively shallow and the habitats themselves can impede net movement, a length of this size may be impractical due to snagging and wave action. Instead, a length of 3 m or less conforms to the dimensions commonly adopted by manufacturers, and emerged as the most prevalent net length observed in this review. Retaining the above dimensions for future sampling practices would facilitate comparability among datasets.

Despite some adapted manta trawl designs capable of being towed at 8 knots, most manta and neuston nets have a maximum tow speed of 3 knots (Gago et al. 2018). With the exception of one study towing at 5 knots, the majority that specified tow speeds operated between 2 and 3 knots (53%). Using slower tow speeds of 1–2 knots is likely to minimise disruption to mangrove and seagrass ecosystems, allowing greater inter-comparability when other net types (e.g. plankton) have to be used. Furthermore, using slower tow speeds would allow smaller mesh sizes to be used, providing a more accurate microplastic yield (Lindeque et al. 2020). A set tow-time cannot be recommended in these ecosystems, as this would be largely dependent on the size of the seagrass meadow or mangrove range, as well as the presence of algal blooms or other eutrophic conditions. If researchers specify the net dimensions, towing speed, towing duration, and the number of replicates, the volume of water sampled can be determined, allowing for a comparison of microplastic abundance per litre across different studies.

Microplastics have a highly variable distribution both within and between ecosystems. Of the studies in this review that used a boat to tow a net, only 1 replicate was used in 62% of studies. In most environmental studies, a minimum of 3 replicates should be used over the area of interest as a compromise between feasibility and capturing a representative sample. Comparison between replicates can also provide information on microplastic variability within a given site (NOAA 2015). The use of water samplers or glass jars provides small sample sizes which may give microplastic abundance results that are unrepresentative of the broader habitat area. Collecting samples deep in mangrove forests or in very shallow areas often practically dictates that these small sample size collection methods are necessary. In these situations, a higher number of replicates would provide a more representative estimation of microplastic abundance, with a minimum of four 0.5–1 L replicates per area (Prata et al. 2020). In addition, when sampling, researchers should take into consideration that the number of replicates required to accurately predict microplastic distribution in an area likely increases as the level of contamination decreases (Bäuerlein et al. 2023; Yu & Flury 2021).

NOAA (2015) and the MSFD technical group (MSFD Technical Group on Marine Litter 2023; MSFD technical subgroup 2013) guidelines recommend that GPS be used to record the starting and stopping points of each trawl, and a flowmeter placed at the aperture of the net to identify sample volume to allow for effective, harmonised, and reproducible methods. This information alongside net dimensions allows researchers to identify the number of microplastics per litre. For net sampling, it is important that researchers always state mesh and aperture size, length, tow speeds, and time towed. For all sampling methods, replicates and volume sampled should be recorded, and ideally a detailed description of equipment used and justification for their choices.

### Sediment sampling in seagrass and mangrove ecosystems

#### Comparisons with current governmental and institutional guidelines

The MSFD technical group (MSFD Technical Group on Marine Litter 2023; MSFD technical subgroup 2013), NOAA (2015), and JPI Oceans BASEMAN group (Frias et al. 2018) have also developed various recommendations for sampling microplastics present in sediment. These methods were originally used to capture sediment for analysis of organic content, benthic organisms, trace metals, and contaminants, and are now also used for microplastic assessment. These recommendations are split into intertidal and subtidal locations.

##### Intertidal

Sampling within the tide lines is inaccessible to most vessels; therefore, heavy machinery such as grabs and corers may be impractical. Consequently, different methods are recommended by agencies and commissions for gathering media at intertidal locations compared to subtidal environments. The MSFD technical subgroup (2013) recommends samples be collected from the top 5 cm of the sediment, as most studies they reviewed sampled at this depth. They also state separate samples should be collected for two different microplastic size classes (20 µm–1 mm and 1–5 mm). For the 20 µm–1 mm size class, they suggest a 15-mL metal spoon to be used to collect approximately 250 mL from the first 5 cm of the sediment. After this, for the 1–5 mm size class, a metal trowel or spoon should collect the top 5 cm of sediment from within a 50 × 50 cm quadrat. They also recommend a minimum of 5 replicates separated by at least 5 m to collect a representative sample. Similarly, the JPI Oceans BASEMAN group (Frias et al. 2018) also recommends sampling the top 5 cm of the sediment. However, in contrast, they suggest collecting 4.5 L from within a 30 × 30 cm quadrat using a metal shovel, with a minimum of 3 replicates across a 100 m transect parallel to the shoreline. NOAA (2015) suggests a wet weight collection of 400 g by corer or grab sampler, followed by drying, weighing, and applying formulae to calculate the mass of total solids.

##### Subtidal

Sampling below the water’s surface typically poses additional complications due to greater inaccessibility. Modifications to sampling methods and the use of snorkelling, SCUBA, or vessels may be necessary. The MSFD technical group (MSFD Technical Group on Marine Litter 2023; MSFD technical subgroup 2013) recommends corer or grab-based approaches (e.g. Van Veen grab, box corer, Gemax corer, Kajak corer), ensuring the seabed remains relatively undisturbed. They recommend sampling to a depth of 5 cm, and once recovered a subsample of approximately 250 mL should be taken to represent each replicate. The JPI Oceans BASEMAN group (Frias et al. 2018) recommends the use of a box corer such as a Reineck corer over other grabs and corers, as it reduces surface deformation while preserving sediment integrity. In addition, they state a minimum of 6 replicate samples should be collected per site for representative estimates.

In summary, the majority of governmental and institutional guidelines for sediment sampling for microplastics recommend the use of shovels/spoons (intertidally) and grabs (subtidally), both to a depth of 5 cm. In this systematic review, shovels/spoons accounted for 44% of the equipment utilised to sample sediment for microplastics, while grabs accounted for only 13%. In addition, 5 cm was the most common sampling depth when researchers used shovels/spoons, grabs, and corers. However, this only accounted for 5% of the total depths sampled by this equipment, with depths ranging from 1 to 170 cm. The method sampling variability in sediment is similar to that seen in surface waters (Fig. 3). To inform choices for future standardised sampling in seagrasses and mangroves, the advantages and disadvantages of each sampling type observed for microplastic collection in sediment are summarised in Table 8.

**Table 8:** Equipment-specific advantages and disadvantages of sediment sampling methods used in seagrass meadows and mangroves. Adapted from Prata et al. (2019)

Equipment	Advantages	Disadvantages
Corers	Versatile, with a range of designs and prices Can be used both intertidally and subtidally Can be deployed by hand or off boat Can obtain depth profiles for each core Minimal surface deformation Maximal sediment retention and integrity Consistent sample sizes	Smaller sample yield than comparative grabs Some designs require boat Possible contamination if using plastic options
Grabs	Ease of use for subtidal sampling Less expensive than comparable corers Usually made of steel, no contamination	Usually requires a boat High risk of surface deformation Difficulty using for shallow, intertidal sampling Cannot obtain any depth profiles Debris may impact sample retention by grab not closing Uneven sample sizes
Shovels/spoons/jars	Inexpensive Easy, rapid sampling Usually stainless steel/glass, no contamination	High risk of surface deformation Difficult to use for subtidal sediment collection Uneven sample sizes

#### Standardising future sediment sampling

Taking into consideration the different advantages and disadvantages of each type of sediment collection equipment (Table 8), protocol suggestions toward more standardised microplastic sampling practices in seagrass meadow and mangrove sediment are given to encourage sampling precision and comparability.

Corers are recommended over other methods for microplastic sampling at both intertidal and subtidal locations in seagrass and mangrove ecosystems. This is firstly due to corers being the most effective equipment for sediment retention, and thus maintain a consistent sample size over multiple replicates (Frias et al. 2018). They also minimally impact surface deformation compared to similar sized grabs, and so cause the least amount of destruction to the target ecosystem. As sediment integrity is maintained, researchers can sample different depth layers to potentially determine contamination over time. Moreover, due to their myriad of designs, corers can be chosen to suit specific locations or budget restrictions.

As sediment depth is a key factor influencing microplastic concentration (Besley et al. 2017), an agreed-upon standard depth would allow for direct comparisons. Unless researchers are specifically sampling multiple layers, a depth of 5 cm is recommended. Microplastic contamination is highly ubiquitous within the superficial layer of marine sediment (top 2.5 cm); therefore to accurately quantify the standing stock of microplastics, cores need to be at least 5 cm (Martin et al. 2017). Five centimetres is also recommended by the MSFD technical subgroup (2013) and the JPI Oceans BASEMAN group (Frias et al. 2018), and was the most common sampling depth seen in this review, allowing for greater inter-comparability with previous datasets. Regardless of equipment chosen, researchers should be mindful that enough sediment is collected per replicate to ensure a representative sample is taken. Recommendations from the MSFD technical subgroup, NOAA (2015), and the JPI Oceans BASEMAN group (Frias et al. 2018) range widely from 250 mL to 12.5 L per replicate. However, Besley et al. (2017) state that a minimum of 11 samples are needed per 100 m of beach to accurately estimate microplastic concentration at a 90% confidence level. It is also evident that microplastics are not homogenously distributed across intertidal and subtidal sediments (Hanvey et al. 2017), and results can vary extensively even within a close geographic distance. Therefore, smaller core sizes combined with a greater number of replicates (minimum 11 per 100 m of transect) may maintain an accurate representation of microplastic abundance while reducing physical strain and time spent on laboratory processing and analysis. Using transects will ensure the distance between replicates is even, non-biased, and repeatable, with a minimum distance of at least 5 m between replicates (MSFD technical subgroup 2013). Samples should not be collected on high or low tide lines unless they are areas of specific interest, as these accumulate a greater proportion of microplastics resulting in overestimation (Hanvey et al. 2017). As with water sampling, researchers should record the GPS coordinates of the start and end points of their sampling transects, and whether the site is intertidal or subtidal.

To enable a higher degree of standardisation between researchers, sediment should be noted by dry weight, as the variable water retention of different sediment types can impact weight. The drying temperature should remain below 60 °C so that plastic polymer integrity is not compromised (Thomas et al. 2020). Recording sediment by dry weight is also recommended by NOAA (2015).

### Future sampling recommendations and ensuring sample integrity

#### Future sampling recommendations in seagrass and mangrove ecosystems

No current microplastic sampling method is ideal for all sampling scenarios, and to date there are a lack of studies directly comparing method efficacy against known quantities of microplastics, which would better enable us to decide on conclusive best-practice sampling methods for surface water and sediment. Until such studies are conducted, improvements can be made by the microplastics research community adopting standardised sampling methodologies and protocols to enable the most direct comparisons between datasets. Based on this systematic review, the suggestions below are provided to aid researchers to reach a consensus for future standardised sampling practices in seagrass meadows and mangroves (Table 9).

**Table 9:** Summary of surface water and sediment sampling protocol suggestions to aid in standardising microplastics sampling in seagrass meadows and mangroves

	Standardisation suggestions based on the reviewed literature
Water sampling	Use a manta trawl Mesh size of 100 µm or below Net dimensions: (60 cm aperture), (25 cm depth), (up to 3 m net length) Tow speeds of 1–2 knots Minimum of 3 replicates per area
	Deploy nets from the side of the vessel out of the ship’s wake Record GPS coordinates at the start and end of each trawl Place flowmeter at net aperture to identify sample volume Net thoroughly rinsed with filtered water before and after each sampling event Storage in non-plastic container (i.e. aluminium bags, glass) Take three environmental blank samples Net sampling—state mesh and aperture size, length, tow speeds, and tow duration Containers—compensate with a higher number of replicates (minimum four 0.5–1 L replicates per area) Report samples in microplastics per litre Always record equipment used, replicates, and volume sampled to make comparisons possible
Sediment sampling	Corers in both intertidal and subtidal locations Stainless steel corer options should be used when possible A core depth of 5 cm, unless researching multiple depth layers Transects to uniformly distance replicates, with at least 5 m between replicates ≥ 11 replicates per 100 m of transect Avoid collecting samples on high or low tide lines, unless in areas of specific interest Record GPS coordinates at the start and end of each sampling transect Note sediment by dry weight per gram or kilogram (dry below 60 °C) If collecting multiple media types (i.e. sediment, water, biota), sediment samples should be collected last Always record equipment used, replicates, and dry weight to make comparisons possible

#### Ensuring sample integrity

In addition to standardising microplastic sampling practices, it is also essential to implement standardised measures to mitigate cross-contamination when collecting samples to accurately report findings. For net sampling, these should be deployed from the side of the vessel out of the ship’s wake to avoid turbulence and contamination from the ship itself (MSFD technical subgroup 2013). Before and after each sampling event, the entirety of the net should be thoroughly rinsed with filtered water, and content from the cod-end of each sample should be stored in a non-plastic container. For sediment sampling, stainless steel options should be chosen over other common materials (e.g. PVC) where possible to avoid plastic contamination. Moreover, if collecting multiple media types (i.e. sediment, water, biota), sediment samples should be collected last, as disturbance to the sediment may cause microplastics to become resuspended in the water, producing inaccurate results from other collected samples. Environmental blanks should always be taken to assess environmental contamination outside the scope of the media sampled. This may include fibres from sampling nets, typically made from nylon or polypropylene, which have been detected in environmental blank samples (Hidalgo-Ruz et al. 2012; Mu et al. 2019). Other sources of contamination may include airborne pollution or contamination from researchers’ vessels, equipment, or clothing. This should be conducted by processing three additional sampling containers filled with filtered water (Pasquier et al. 2022).

## Conclusions

This review has highlighted the inconsistencies in surface water and sediment microplastic sampling methodologies used in economically and environmentally important mangroves and seagrasses. In these ecosystems, the most widely adopted surface water microplastic sampling methods are plankton nets, which is misaligned with current institutional and governmental guidelines, as no group endorses the use of plankton nets. For sediment sampling, shovels/spoons were the most utilised method. While conforming to certain guidelines, this represented less than half of the sediment sampling methods used in these ecosystems. The clear disparities in methods used highlight an urgent need for consensus on best sampling practices. This study advises the use of manta nets equipped with 100 µm meshes, and sediment corers with sampling depth standardised at 5 cm, alongside a set of other recommendations (Table 9). These standardised protocols could greatly enhance the comparability of data across studies, allowing for more robust assessments of microplastic pollution in mangroves and seagrass meadows. By adhering to standardised sampling protocols, researchers could provide a more cohesive global understanding of the scale of plastic pollution in these ecosystems, which would lay the foundation for strategic prevention and remediation solutions for environmental managers.

## Supplementary Information

Below is the link to the electronic supplementary material.

Supplementary file1 (DOCX 29.5 KB)

## Data Availability

All articles used in this review are available in the supplementary material (Table S1).
