# Supplementary material for: A systematic review to assess current surface water and sediment microplastic sampling practices in seagrass and mangrove ecosystems
Source: Environ Sci Pollut Res Int. 2024 Dec 11;31(59):66615–29. doi: 10.1007/s11356-024-35690-9 (PMC11666669; doi:10.1007/s11356-024-35690-9)
Supplement: Supplementary file 1 — Supplementary file1 (DOCX 29.5 KB) [file 11356_2024_35690_MOESM1_ESM.docx]

*Supplementary Information for:*

**A systematic review to assess current surface water and sediment microplastic sampling practices in seagrasses and mangrove ecosystems**

Jack Greenshields^a^, Amie Anastasi^ab^, Andrew D. Irving^ab^, Angela Capper^a^
^a^ Coastal Marine Ecosystems Research Centre, Central Queensland University, Gladstone, Australia, 4680
^b^ Central Queensland Innovation and Research Precinct, Central Queensland University, Rockhampton, Australia, 4701

**Table S1.** Citations for all articles used in the systematic review.

| *Aguirre-Sanchez et al. (2024)* | Prevalence of microplastics in Peruvian mangrove sediments and edible mangrove species |
| --- | --- |
| *Aliabad et al. (2019)* | Microplastics in the surface seawaters of Chabahar Bay, Gulf of Oman (Makran Coasts) |
| *Al-Tarshi et al. (2024)* | Marine litter and microplastic pollution in mangrove sediments in the Sea of Oman |
| *Alves et al. (2019)* | Microplastic in the sediments of a highly eutrophic tropical estuary |
| *Azhari et al. (2022)* | Microplastic accumulation in various sizes of Nerita articulata A. Gould, 1847 snails in the mangrove area of Batukaras Pangandaran, West Java, Indonesia |
| *Barasarathi et al. (2014)* | Microplastic Abundance in Selected Mangrove Forest in Malaysia |
| *Bonifacio et al. (2022)* | Microplastic in Sediments and Ingestion Rates in Three Edible Bivalve Mollusc Species in a Southern Philippine Estuary |
| *Boshoff et al. (2023)* | The role of seagrass meadows in the accumulation of microplastics: Insights from a South African estuary |
| *Bulleri et al. (2021)* | The sea cucumber Holothuria tubulosa does not reduce the size of microplastics but enhances their resuspension in the water column |
| *Celis-Hernandez et al. (2021)* | Microplastic distribution in urban vs pristine mangroves: Using marine sponges as bioindicators of environmental pollution |
| *Chaisanguansuk et al. (2023)* | Preliminary study on microplastic abundance in mangrove sediment cores at Mae Klong River, upper Gulf of Thailand |
| *Chaisanguansuk et al. (2023)* | Microplastic Contamination in the Coastal Environment: A Case Study from the Mae Klong Estuary, Samut Songkhram |
| *Chen & Lee (2021)* | Contribution of Microplastics to Carbon Storage in Coastal Wetland Sediments |
| *Cheng et al. (2021)* | A baseline for microplastic particle occurrence and distribution in Great Bay Estuary |
| *Chico-Ortiz et al. (2020)* | Microplastics in Ghanaian coastal lagoon sediments: Their occurrence and spatial distribution |
| *Compa et al. (2022)* | Spatial distribution of macro- and micro-litter items along rocky and sandy beaches of a Marine Protected Area in the western Mediterranean Sea |
| *Cordova et al. (2021)* | Characterization of microplastics in mangrove sediment of Muara Angke Wildlife Reserve, Indonesia |
| *Cordova et al. (2023)* | Microplastics leaving a trace in mangrove sediments ever since they were first manufactured: A study from Indonesia mangroves |
| *Cozzolino et al. (2020)* | Species-specific plastic accumulation in the sediment and canopy of coastal vegetated habitats |
| *da Costa et al. (2023)* | Abundance, composition, and distribution of microplastics in intertidal sediment and soft tissues of four species of Bivalvia from Southeast Brazilian urban beaches |
| *Dahl et al. (2021)* | A temporal record of microplastic pollution in Mediterranean seagrass soils |
| *Deng et al. (2020)* | Microplastics and accumulated heavy metals in restored mangrove wetland surface sediments at Jinjiang Estuary (Fujian, China). |
| *Ding et al. (2022)* | Distribution and retention of microplastics in plantation mangrove forest sediments |
| *Dou et al. (2021)* | Microplastics on beaches and mangrove sediments along the coast of South China |
| *Duan et al. (2021)* | How mangrove plants affect microplastic distribution in sediments of coastal wetlands: Case study in Shenzhen Bay, South China |
| *Dung et al. (2021)* | Depth Profiles of Microplastics in Sediment Cores from Two Mangrove Forests in Northern Vietnam |
| *Exposito et al. (2021)* | Microplastics levels, size, morphology and composition in marine water, sediments and sand beaches. Case study of Tarragona coast (western Mediterranean) |
| *Fagiano et al. (2023)* | Breaking the paradigm: Marine sediments hold two-fold microplastics than sea surface waters and are dominated by fibers |
| *Fagiano et al. (2023)* | The hyperbenthic environment: A forgotten habitat for plastic pollution |
| *Fatema et al. (2023)* | Microplastic pollution in surface waters and sediments matrices of the Sundarbans – The largest single block of tidal halophytic mangrove forest in the world |
| *Fitri & Patria (2019)* | Microplastic contamination on Anadara granosa Linnaeus 1758 in Pangkal Babu mangrove forest area, Tanjung Jabung Barat district, Jambi |
| *Fitri & Patria (2019)* | Microplastic contamination on Cerithidea obtusa (Lamarck 1822) in Pangkal Babu Mangrove Forest Area, Tanjung Jabung Barat District, Jambi. |
| *Fitri & Patria (2021)* | Microplastic in mangrove horn snail Telescopium telescopium (Linnaeus, 1758) at mangrove ecosystem, Rambut Island, Jakarta Bay, Indonesia. |
| *Garcés-Ordóñez et al. (2019)* | Marine litter and microplastic pollution on mangrove soils of the Ciénaga Grande de Santa Marta, Colombian Caribbean |
| *Garces-Ordonez et al. (2021)* | Abundance, distribution, and characteristics of microplastics in coastal surface waters of the Colombian Caribbean and Pacific |
| *Garcés-Ordóñez et al. (2023)* | Seasonal variation in plastic litter pollution in mangroves from two remote tropical estuaries of the Colombian Pacific |
| *Govender et al. (2020)* | Towards Characterising Microplastic Abundance, Typology and Retention in Mangrove-Dominated Estuaries |
| *Hamid et al. (2020)* | Microplastics abundance and uptake by meretrix lyrata (Hard clam) in mangrove forest. |
| *Hanifah et al. (2018)* | Abundance of Microplastics in the Water and Mangrove Crab Metopograpsus quadridentata in Pramuka Island, Jakarta |
| *Hu et al. (2022)* | Distribution characteristics of microplastics in the soil of mangrove restoration wetland and the effects of microplastics on soil characteristics |
| *Hu et al. (2023)* | Sediment and interstitial water heavy metals in mangrove restoration wetland and preliminary exploration of microplastics in interstitial water |
| *Huang et al. (2020)* | Seagrass beds acting as a trap of microplastics - Emerging hotspot in the coastal region? |
| *Huang et al. (2021)* | New Insights into the Microplastic Enrichment in the Blue Carbon Ecosystem: Evidence from Seagrass Meadows and Mangrove Forests in Coastal South China Sea |
| *Ibrahim et al. (2021)* | Spatiotemporal microplastic occurrence study of Setiu Wetland, South China Sea |
| *Idris et al. (2023)* | Microplastic contamination in sediment and Strombus sp. on Bintan Island, Indonesia |
| *Jeyasanta et al. (2020)* | Occurrence and characteristics of microplastics in the coral reef, sea grass and near shore habitats of Rameswaram Island, India |
| *Jeyasanta et al. (2023)* | Microplastic pollution and its implicated risks in the estuarine environment of Tamil Nadu, India |
| *Jiao et al. (2022)* | Mangrove forest: An important coastal ecosystem to intercept river microplastics |
| *Jitrapat et al. (2024)* | Ingestion and adherence of microplastics by estuarine mysid shrimp |
| *Jittalerk & Babel (2024)* | Microplastic contamination in Thai vinegar crabs (Episesarma mederi), giant mudskippers (Periophthalmodon schlosseri), and their surrounding environment from the Bang Pu mangrove forests, Samut Prakan province, Thailand |
| *Johnson et al. (2023)* | Microplastic abundance in urban vs. peri-urban mangroves: The feasibility of using invertebrates as biomonitors of microplastic pollution in two mangrove dominated estuaries of southern Africa |
| *Jones et al. (2020)* | Microplastic accumulation in a Zostera marine L. bed at Deerness Sound, Orkney, Scotland |
| *Jong et al. (2022)* | Microplastics in equatorial coasts: Pollution hotspots and spatiotemporal variations associated with tropical monsoons |
| *Kama et al. (2021)* | Microplastic concentration in column seawater compartment in Burau, Luwu Regency, South Sulawesi, Indonesia |
| *Kannankai et al. (2022)* | Urban mangrove ecosystems are under severe threat from microplastic pollution: a case study from Mangalavanam, Kerala, India |
| *Karthik et al. (2022)* | Microplastic pollution in fragile coastal ecosystems with special reference to the X-Press Pearl maritime disaster, southeast coast of India |
| *Kerubo et al. (2021)* | Microplastics pollution in the sediments of creeks and estuaries of Kenya, western Indian Ocean |
| *Khuyen et al. (2022)* | Assessing Microplastic Prevalence and Dispersion from Saigon Urban Canals via Can Gio Mangrove Reserve to East Sea by Raman Scattering Microscopy |
| *Kreitsberg et al. (2021)* | Seagrass beds reveal high abundance of microplastic in sediments: A case study in the Baltic Sea |
| *Kumkar et al. (2021)* | Big eyes can't see microplastics: Feeding selectivity and eco-morphological adaptations in oral cavity affect microplastic uptake in mud-dwelling amphibious mudskipper fish |
| *Ledet et al. (2024)* | Trapping of microplastics and other anthropogenic particles in seagrass beds: Ubiquity across a vertical and horizontal sampling gradient |
| *Li et al. (2018)* | Characterization, source, and retention of microplastic in sandy beaches and mangrove wetlands of the Qinzhou Bay, China |
| *Li et al. (2019)* | Abundance and characteristics of microplastics in the mangrove sediment of the semi-enclosed Maowei Sea of the south China sea: New implications for location, rhizosphere, and sediment compositions |
| *Li et al. (2020)* | Field study of the microplastic pollution in sea snails (Ellobium chinense) from mangrove forest and their relationships with microplastics in water/sediment located on the north of Beibu Gulf. |
| *Li et al. (2020)* | The distribution, characteristics and ecological risks of microplastics in the mangroves of Southern China |
| *Li et al. (2022)* | Mangrove leaves: An undeniably important sink of MPs from tidal water and air |
| *Li et al. (2022)* | Microplastics distribution in different habitats of Ximen Island and the trapping effect of blue carbon habitats on microplastics |
| *Li et al. (2023)* | Distinct microplastics abundance variation in root-associated sediments revealed the underestimation of mangrove microplastics pollution |
| *Linh et al. (2023)* | Contamination of microplastics in mangrove sediment cores from Lach Huyen area, Hai Phong city, Vietnam |
| *Lins-Silva et al. (2021)* | A fresh look at microplastics and other particles in the tropical coastal ecosystems of Tamandaré, Brazil |
| *Liu et al. (2022)* | Ecological interception effect of mangroves on microplastics |
| *Maghsodian et al. (2021)* | Microplastics accumulation in sediments and Periophthalmus waltoni fish, mangrove forests in southern Iran |
| *Martin et al. (2020)* | Exponential increase of plastic burial in mangrove sediments as a major plastic sink. |
| *Martinez et al. (2024)* | The power of Posidonia oceanica meadows to retain microplastics and the consequences on associated macrofaunal benthic communities |
| *Maulida et al. (2024)* | Analysis of types, forms and abundance of microplastics in the mangrove forest area of pusong island, langsa city |
| *Mohamed et al. (2023)* | Depth Profiles of Microplastic in Sediment Cores in the Mangrove Area of Kuala Gula Mangrove, Malaysia |
| *Moniuszko et al. (2023)* | Accumulation of Plastics and Trace Elements in the Mangrove Forests of Bima City Bay, Indonesia. |
| *Nabizadeh et al. (2019)* | Microplastic pollution on the Persian Gulf shoreline: A case study of Bandar Abbas city, Hormozgan Province, Iran |
| *Nafisyah et al. (2023)* | Microplastic abundance in Surabaya mangrove areas during the wet season |
| *Nahian et al. (2022)* | Occurrence, spatial distribution, and risk assessment of microplastics in surface water and sediments of Saint Martin Island in the Bay of Bengal |
| *Nair et al. (2021)* | Seasonal microplastic documentation in Kerala mangrove sediments |
| *Naji et al. (2017)* | The occurrence of microplastic contamination in littoral sediments of the Persian Gulf, Iran. |
| *Naji et al. (2019)* | Small microplastic particles (S-MPPs) in sediments of mangrove ecosystem on the northern coast of the Persian Gulf. |
| *Navarrete-Fernandez et al. (2022)* | The role of seagrass meadows in the coastal trapping of litter |
| *Navarro et al. (2022)* | Unraveling Microplastic Pollution in Mangrove Sediments of Butuan Bay, Philippines |
| *Nawar et al. (2023)* | Characterization of microplastic pollution in the Pasur river of the Sundarbans ecosystem (Bangladesh) with emphasis on water, sediments, and fish. |
| *Neelavannan et al. (2023)* | Microplastics in the Ganga-Brahmaputra delta: Sources and Pathways to the Sundarbans Biosphere Reserve - an UNESCO World Heritage Centre |
| *Ni'am et al. (2022)* | Microplastics in Sediments of East Surabaya, Indonesia: Regional Characteristics and Potential Risks |
| *Nor & Obbard (2014)* | Microplastics in Singapore’s coastal mangrove ecosystems |
| *Paes et al. (2022)* | Widespread microplastic pollution in mangrove soils of Todos os Santos Bay, northern Brazil |
| *Pan et al. (2020)* | Riverine microplastic pollution matters: A case study in the Zhangjiang River of Southeastern China. |
| *Pan et al. (2023)* | Reinforced human intervention drives microplastic pollution in estuarine beaches and nearshore sediments of Dongshan Bay, China |
| *Pariatamby et al. (2020)* | Status of microplastic pollution in aquatic ecosystem with a case study on cherating river, Malaysia. |
| *Plee & Pomory (2020)* | Microplastics in sandy environments in the Florida Keys and the panhandle of Florida, and the ingestion by sea cucumbers (Echinodermata: Holothuroidea) and sand dollars (Echinodermata: Echinoidea) |
| *Pradit et al. (2022)* | The Occurrence of Microplastics in Sediment Cores from Two Mangrove Areas in Southern Thailand |
| *Prarat et al. (2024)* | Microplastic occurrence in surface sediments from coastal mangroves in Eastern Thailand: Abundance, characteristics, and ecological risk implications |
| *Pricilla et al. (2019)* | Microplastic abundance in the water, seagrass, and sea hare Dolabella auricularia in Pramuka Island, Seribu Islands, Jakarta Bay, Indonesia |
| *Ragoobur et al. (2023)* | Microplastics in estuarine water and sediment in Mauritius |
| *Rahmawati & Patria (2019)* | Microplastics Dissemination from Fish Mugil dussumieri and Mangrove Water of Muara Teluknaga, Tangerang, Banten |
| *Ramili & Umasangaji (2022)* | Accumulation of Microplastics (MPs) Sedimentary in Seagrass Meadows on Mare Island Conservation Area, North Maluku, Indonesia |
| *Rasyid et al. (2022)* | Assessment of marine debris in seagrass beds of Pramuka Island, Kepulauan Seribu |
| *Rios-Fuster et al. (2023)* | Are seafloor habitats influencing the distribution of microplastics in coastal sediments of a Marine Protected Area? |
| *Rose & Webber (2019)* | Characterization of microplastics in the surface waters of Kingston Harbour |
| *Ruangpanupan et al. (2022)* | Microplastics in the surface seawater of Bandon Bay, Gulf of Thailand |
| *Sawalman et al. (2021)* | Microplastic abundance in sea urchins (Diadema setosum) from seagrass beds of Barranglompo Island, Makassar, Indonesia |
| *Seeruttun et al. (2023)* | Mangrove and microplastic pollution: A case study from a small island (Mauritius) |
| *Shelciya et al. (2023)* | Preliminary Study on the Role of Mangroves in Entrapping Microplastics in Tuticorin Coast of Gulf of Mannar, Southeast Coast of India |
| *Shylaja et al. (2021)* | Assessment of non-degradable litter and its impact on the benthic community of selected mangrove ecosystems of Kerala, India |
| *Sulistiowati et al. (2021)* | The distribution of marine debris and microplastic in Tidung Kecil Island, Jakarta Bay and Sembilang National Park, Palembang |
| *Supriatna et al. (2023)* | Microplastics contaminant in Telescopium telescopium (gastropods), the keystone mangrove species and their habitat at brackish water pond, East Java, Indonesia |
| *Sutega et al. (2021)* | Spatial and temporal distribution of microplastic in surface water of tropical estuary: Case study in Benoa Bay, Bali, Indonesia |
| *Tahir et al. (2019)* | Studies on microplastic contamination in seagrass beds at Spermonde Archipelago of Makassar Strait, Indonesia |
| *Tahir et al. (2020)* | Microplastic assessment in Seagrass ecosystem at Kodingareng Lompo Island of Makassar City. |
| *Tan et al. (2023)* | Abundance and distribution of microplastics in tropical estuarine mangrove areas around Penang, Malaysia |
| *Trindade et al. (2023)* | Microplastics in surface waters of tropical estuaries around a densely populated Brazilian bay |
| *Tubagus et al. (2020)* | Identification of Microplastic Composition on Clams (Gafrarium tumidum) and Sediments in Pari Island, Seribu Islands, Jakarta |
| *Utami et al. (2021)* | Microplastics as a sedimentary component in reef systems: A case study from the Java Sea |
| *Valsan et al. (2024)* | Seasonal variation of microplastics in tropical mangrove waters of South-western India |
| *Valsan et al. (2024)* | Spatiotemporal variations of microplastics in the surface sediments of a tropical mangrove ecosystem in south-western India |
| *Vandale et al. (2022)* | Monitoring microplastics in Tampa Bay |
| *Wang et al. (2020)* | Microplastic pollution in intertidal sediments along the coastline of Chin |
| *Wang et al. (2022)* | High Microplastic Contamination in Juvenile Tri-Spine Horseshoe Crabs: A Baseline Study of Nursery Habitats in Northern Beibu Gulf, China |
| *Wei et al. (2022)* | Microplastic Distribution and Influence Factor Analysis of Seawater and Surface Sediments in a Typical Bay With Diverse Functional Areas: A Case Study in Xincun Lagoon, China |
| *Wicaksono et al. (2021)* | Distribution and Seasonal Variation of Microplastics in Tallo River, Makassar, Eastern Indonesia |
| *Wright et al. (2023)* | Microplastic accumulation in Halophila ovalis beds in the Swan-Canning Estuary, Western Australia |
| *Wu et al. (2022)* | Linking human activity to spatial accumulation of microplastics along mangrove coasts |
| *Xu et al. (2023)* | The role of bio-geomorphic feedbacks in shaping microplastic burial in blue carbon habitats |
| *Yan et al. (2022)* | Microplastic pollution and enrichment of distinct microbiota in sediment of mangrove in Zhujiang River estuary, China |
| *Yang et al. (2023)* | Enrichment and ecological risks of microplastics in mangroves of southern Hainan Island, China |
| *Yona et al. (2019)* | Microplastics in the surface sediments from the eastern waters of Java Sea, Indonesia |
| *Yoswaty et al. (2021)* | The Threat of Microplastic Waste in Dumai Waters, Province of Riau, Indonesia |
| *Yu et al. (2022)* | Distribution, characteristics, and human exposure to microplastics in mangroves within the Guangdong-Hong Kong-Macao Greater Bay Area |
| *Yu et al. (2023)* | Vertical distribution, accumulation, and characteristics of microplastics in mangrove sediment in China |
| *Zamprogno et al. (2021)* | Spatial distribution of microplastics in the superficial sediment of a mangrove in Southeast Brazil: A comparison between fringe and basin |
| *Zandhi et al. (2019)* | Conditions for Sediment Coating Microplastic in Mangrove Ecosystems in Kupang and Rote, East Nusa Tenggara, Indonesia |
| *Zhang et al. (2020)* | Dynamic distribution of microplastics in mangrove sediments in Beibu Gulf, South China: Implications of tidal current velocity and tidal range. |
| *Zhang et al. (2022)* | Tidal variation shaped microplastic enrichment patterns in mangrove blue carbon ecosystem of northern Beibu Gulf, China |
| *Zhang et al. (2024)* | Human activities altered the enrichment patterns of microplastics in mangrove blue carbon ecosystem in the semi-enclosed Zhanjiang Bay, China |
| *Zhao et al. (2022)* | Eelgrass (Zostera marina) and its epiphytic bacteria facilitate the sinking of microplastics in the seawater |
| *Zhou et al. (2020)* | Characteristics and distribution of microplastics in the coastal mangrove sediments of China |
| *Zuo et al. (2020)* | Microplastics in mangrove sediments of the Pearl River Estuary, South China: Correlation with halogenated flame retardants’ levels. |
